# Identification and validation of a novel prognosis model based on m5C-related long non-coding RNAs in colorectal cancer

**DOI:** 10.1186/s12935-023-03025-2

**Published:** 2023-09-05

**Authors:** Ziyang Di, Gaoran Xu, Zheyu Ding, Chengxin Li, Jialin Song, Guoquan Huang, Jinsen Zheng, Xinyao Zhang, Bin Xiong

**Affiliations:** 1 Department of Gastrointestinal Surgery, Zhongnan Hospital of Wuhan University, Wuhan, China; 2 Hubei Key Laboratory of Tumor Biological Behaviors, Wuhan, China

**Keywords:** CRC, m5C, lncRNA, Tumor immune microenvironment, Risk model

## Abstract

**Background:**

The prognosis of tumor patients can be assessed by measuring the levels of lncRNAs (long non-coding RNAs), which play a role in controlling the methylation of the RNA. Prognosis in individuals with colorectal adenocarcinoma (CRC) is strongly linked to lncRNA expression, making it imperative to find lncRNAs that are associated with RNA methylation with strong prognostic value.

**Methods:**

In this study, by analyzing TCGA dataset, we were able to develop a risk model for lncRNAs that are associated with m5C with prognostic significance by employing LASSO regression and univariate Cox proportional analysis. There were a number of methods employed to ensure the model was accurate, including multivariate and univariate Cox regression analysis, Kaplan analysis, and receiver operating characteristic curve analysis. The principal component analysis, GSEA and GSVA analysis were used for risk model analysis. The CIBERSORT instrument and the TIMER database were used to evaluate the link between the immune cells that infiltrate tumors and the risk model. In vitro experiments were also performed to validate the predicted m5C-related significant lncRNAs.

**Results:**

The m5c regulators were differentially expressed in colorectal cancer and normal tissue. Based on the screening criteria and LASSO regression, 11 m5c-related lncRNAs were identified for developing the prognostic risk model. Multivariate and univariate Cox regression analysis showed the risk score is a crucial prognostic factor in CRC patients. The 1-year, 3-year, and 5-year AUC curves showed the risk score was higher than those identified for other clinicopathological characteristics. A nomogram using the risk score as a quantitative tool was developed for predicting patients' outcomes in clinical settings. In addition, the risk profile of m5C-associated lncRNAs can discriminate between tumor immune cells’ characteristics in CRC. Mutation patterns and chemotherapy were analyzed between high- and low- risk groups of CRC patients. Moreover, TNFRSF10A-AS1 was chosen for the in vitro verification of the m5C-connected lncRNA to demonstrate impressive effects on the proliferation, migration and invasion of CRC cells.

**Conclusion:**

A risk model including the prognostic value of 11 m5C-associated lncRNAs proves to be a useful prognostic tool for CRC and improves the care of patients suffering from CRC based on these findings.

**Supplementary Information:**

The online version contains supplementary material available at 10.1186/s12935-023-03025-2.

## Backgroud

According to the Global Cancer Statistics 2020, CRC’s morbidity ranks 3rd in the world, and the death rate ranks 2nd [1]. Currently, the major treatment approaches are surgery, endoscopic resection, neoadjuvant chemotherapy, adjuvant chemotherapy, radiotherapy, targeted therapy, etc. [2]. Killing tumor cells as much as possible to minimize the number of tumor cells is the ultimate goal of tumor treatment, thereby boosting the patients’ PFS (progression-free survival), and OS (overall survival), and improving patient prognosis [3]. However, the current status of colorectal cancer treatment is not optimistic, with the characteristics of a high metastasis rate, high recurrence rate, and high drug resistance rate [4]. Therefore, searching for molecular markers related to the prognosis of colorectal cancer, as well as diagnostic and therapeutic targets based on tumor molecular markers, will definitely have basic theoretical significance and important clinical guidance value [5].

Epigenetics is the study of reversible and heritable phenotypes, including RNA methylation, DNA methylation, noncoding RNA alterations, histone modifications, and chromatin rearrangements. Currently, more than a hundred different chemical alterations to RNA have been reported [6–9]. This field of study, known as epigenetics, is rapidly expanding. The methylation of adenine at its N6 position was first identified in mRNA in 1974. The m6a base modification is the most common type of internal alteration seen on eukaryotic mRNA [10]. Internal mRNA modifications have been studied continuously over the past 5 decades. The disclosed mRNA modifications include, but are not limited to, inosine (I), N1-methyladenosine (m1A), uridine (U), 5-methylcytosine (m5C), ribose-methylation (2′-O-Me), N6-methyladenosine (m6A), pseudouridine (Ψ), and 5-hydroxymethylcytosine (hm5C) [11, 12]. m5C is a conserved and universal marker of RNA in all domains of life. m5C is found in a wide range of RNAs but is most abundant in eukaryotic tRNA and rRNA [13]. A methyltransferase complex, composed of methyltransferase "writers," demethylase "erasures," and m5C binding protein "readers," catalyzes RNA m5C methylation [14–16]. The translocation, stability, and translation of the target RNA have all been shown to be affected by m5C modifications, which have been shown to affect the progression of cancer [17]. With a specific regulator, RNA m5C can mediates the activation of oncogenic pathways and forms a microenvironment suitable for the migration and metastasis of various cancer cells. For instance, NSUN5 and NSUN6 were reported to be associated with metastasis in skin cancer and breast cancer. The former methylase and the specific reader ALYREF are overexpressed in metastatic stage of head and neck squamous cell carcinoma [18]. Although the latter participates in RNA–protein interactions, an MST1/2-antagonizing lncRNA for YAP activation inhibits the activity of macrophage stimulating 1 (a protein serine kinase) in an NSUN6-dependent manner, which facilitates bone metastasis in breast cancer [19]. In urothelial carcinoma of the bladder, NSUN2 targets the 3′ untranslated region (3′-UTR) and stabilizes the mRNA of HGDF by generating the RNA m5C modification, while the reader YBX1 binds to the m5C region with the help of the partner protein ELAVL1 (an mRNA stability maintainer). The activation of the NSUN2/YBX1/HDGF axis was proven to promote cell growth, tumor progression and metastasis [20]. Recent researches indicate that SUMO-2/3 modification of the RNA methyltransferase NSUN2 enhances the onset and progression of gastric cancer [21]. Notably, NSUN6, identified as a methyltransferase targeting mRNA, may be part of a quality control mechanism involved in translation termination fidelity to regulate tumor development [17]. In view of the impact of m5C RNA methylation on tumor progression, it is necessary to comprehensively analyze it and its related genes.

Researchers have discovered that many factors play a part in colorectal cancer's pathogenesis, including protein-coding genes and non-coding genes. However, the occurrence and evolution of CRC is an intricate regulatory process, and there are still many unknowns that require further research. lncRNA (long non-coding RNA) is a kind of non-coding RNA that is widely distributed in human genes. It can participate in the formation of a complex gene expression regulatory network and regulate various biological processes [22, 23]. Recent research results show that lncRNA can play a vital part in the growth, differentiation, and apoptosis of stem cells [24–27]. In addition, cell proliferation, death, and migration are all influenced by lncRNAs because of their roles in regulating many biochemical pathways, which in turn affects gene expression [28]. lncRNA has been shown to play a significant part in the growth of cancerous tumors [29]. Evidence from many researches suggests that genes involved in m5c methylation regulate the methylation level of lncRNA, thus impacting tumor onset and progression. For example, the RNA methyltransferase NSUN2 is recruited by FOXC2-AS1 to FOXC2 mRNA, elevating its m5C level and boosting its interaction with YBX1 to control gastric cancer onset and progression [30]. Similarly, G3BP1 oncoprotein is recruited by m5C-modified H19 lncRNA, which may similarly enhance hepatocellular carcinoma onset and progression [31]. However, reports about lncRNA on the regulation of m5C methylation are still rare. For this reason, it is crucial to investigate the link between lncRNAs and m5C methylation in cancerous tumors.

A tumor microenvironment (TME) is defined as the complex and rich multicellular environment in which tumors develop. It consists of immune cells, fibroblasts, endothelial cells, and mesenchymal cells that cooperatively mesh and communicate with each other and with the heterogeneous cancer cells themselves [32]. Tumorigenesis and metastasis rely heavily on TME’s interactions with tumor cells [33]. Tumor onset and progression are mostly accounted for by lncRNAs which have been shown to alter the TME (tumor microenvironment) and partake in a significant duty in immune identification and evasion in tumor-infiltrating immune cells [34, 35]. In one study, LINC00662 stimulates the production and release of WNT3A. The Wnt/β-catenin pathway promotes macrophage polarization and cancer cell migration in the TME via autocrine and paracrine mechanisms in macrophages and hepatocellular carcinoma, respectively [36]. Glioblastoma cells release exosomes containing lncRNAs, which are taken up by tumor-associated macrophages (TAM) and used to stimulate microglial M2 polarization. This M2 polarization is linked to the production of C5/C5a, a component of the complement system, which occurs after ENO1 binding and promotes p38 MAPK activation, thus enhancing chemoresistance [37]. However, there are a few ongoing studies on the link between immune cell infiltration and lncRNA in CRC, and more studies are required.

While previous bioinformatics research has focused on RNA alterations, this is a comprehensive examination of the involvement of m5C regulators in CRC. In this investigation, we used m5C-associated lncRNAs expression data from TGCA (The Cancer Genome Atlas) dataset. 11 m5C-associated lncRNAs with prognostic significance were examined, a prognostic signal of m5C-associated lncRNAs was developed, and the association between immune cell infiltration subtypes and m5C-related lncRNAs was further investigated. Our aim was to explore the immune microenvironment for RNA methylation of m5C-associated lncRNA in CRC with different genetic features, impact on tumor, and prognostic value so as to offer CRC management guidance.

## Methods and materials

### Data acquisition and preprocessing

Transcriptome analysis of raw data and corresponding clinical information of the COAD and READ cohort were downloaded from TCGA data portal (http://cancergenome.nih.gov/). The TCGA-COAD and TCGA-READ datasets were searched for mRNA and lncRNA transcriptome sequencing data and associated clinical metadata for 612 subjects, comprising 568 tumor-infested samples and 44 adjoining noncancerous samples. In total, 548 cases of CRC were summarized in Table 1 after excluding the patients for whom we did not have survival data, The lncRNAs from the TCGA dataset were annotated using a file downloaded from the GENCODE website, which included lncRNAs’ annotations in the Genome Reference Consortium Human Build 38 (GRCh38). 13,142 lncRNAs were found in the TCGA dataset by using the gene’s Ensemble IDs as identifiers. Twelve m5C regulators were chosen for subsequent study after reading the existing literature; they are NSUN3, NSUN5, NSUN7, NSUN6, NSUN2, DNMT1, NSUN4, DNMT3A, DNMT3B, TRDMT1, TET2, and ALYREF. We used the "limma" tool in R software to conduct analyses on differentially expressed genes (DEGs). For DEGs, we used a cutoff value of |log2Fold Change|≥ 1 and a significance level of p < 0.05. The DEGs heatmap and vioplot were plotted using the "pheatmap" and "vioplot" packages, respectively. The lncRNAs associated with m5C were screened using the "limma" R program. The association between the 13,162 lncRNAs and the 12 m5C regulators was analyzed using the "cor.test." lncRNAs that were related to m5C regulators and with p-value < 0.001 and |correlation coefficient|> 0.3 were retained for further analysis.

**Table 1:** The clinical characteristics of colorectal adenocarcinoma patients in the TCGA database

Variables	No. of patients	Percentage (%)
Age(yeas)		
≤ 65	237	43.2
> 65	311	56.8
Gender		
Female	256	46.7
Male	292	53.3
Pathological stage		
I	96	17.5
II	210	38.3
III	149	27.2
IV	78	14.2
Unknown	15	2.8
T stage		
T1	16	2.9
T2	96	17.5
T3	373	68.1
T4	63	11.5
N stage		
N0	323	58.9
N1	130	23.7
N3	94	17.2
Unkown	1	0.2
M stage		
M0	408	74.4
M1	77	14.1
Unkown	63	11.5

### Bioinformatic analysis

We developed our protein–protein interaction network using the information available in version 11.0 of the STRING database (http://www.string-db.org). A score of 0.7 or higher on the interaction was necessary (high confidence). Then, we compared the co-expression patterns of different m5C regulators using Pearson's correlation coefficient. R package corrplot v.0.84 was used in the generation of the correlation plot. The univariate Cox regression analysis was conducted to determine the prognostic significance of m5C-related lncRNA. We next calculated the HR, 95% CI and p-value for each m5C-associated lncRNA, with a p-value greater than 0.01 indicating statistical significance. To evaluate the prognostic value of the m5C regulators, we performed univariate Cox regression analysis. The prognostic signature was constructed using the lncRNAs linked to prognosis and a LASSO-penalized Cox regression analysis. Each subject’s risk score was calculated as per the following equation: risk score = coefficient 1 ∗ value1 + coefficient 2 ∗ value 2 + coefficient 3 ∗ value3 + coefficient4 ∗ value 4 + coefficient5 ∗ value5 + coefficient6 ∗ value 6 + coefficient 7 ∗ value7 + coefficient 8 ∗ value 8 + coefficient 9 ∗ value 9 + coefficient 10 ∗ value 10 + coefficient 11 ∗ value 11. The value was the relative expression level of each selected gene, and the lncRNA regression coefficient is denoted by Coefi. The delineation of the high-risk and low-risk categories was done by using the median risk score in the control group. Its predictive ability was tested using a time-dependent ROC and Kaplan–Meier analysis. The testing group's risk category was predicted by the cutoff score used for the training group in an accurate manner. Clinicopathologic features (T stage, tumor stage, age, N stage, and M stage) were tested for independence from the risk signature using both multivariate and univariate Cox regression analysis. A subgroup evaluation was performed to test the signature's viability. The independent prognosis-related parameters gotten from multivariate Cox regression analysis were incorporated into a nomogram to facilitate the implementation of our prognostic model in evaluating the 1-, 3-, and 5-year OS of patients suffering from CRC by clinicians. Nomogram’s prognostic value was confirmed using a c-index and a calibration curve.

### Genomic enrichment analysis (GSEA) and genomic variation analysis (GSVA)

GSEA enrichment analysis was performed using the "clusterProfiler" R package. KEGG (Kyoto Encyclopedia of Genes and Genomes) analyses, downloaded from the MSigDB database, were used as data for performing GSEA analysis in the study. The "GSVA" R package was used to perform GSVA enrichment analysis to obtain results on the differences in signaling pathways between high and low risk groups. The data source was the "Hallmark gene sets" gene sets downloaded from the MSigDB database, *p* < 0.05.

### Predicting chemotherapy response

The chemotherapy reaction profile of CRC patients was predicted by the R package "pRRophetic". The half-maximal inhibitory concentration (IC50) of the samples was calculated by ridge regression. Prediction accuracy was assessed by tenfold cross-validation on the basis of the GDSC training set.

### Estimating the infiltration of TME cells in CRC

The relative abundance of each cell infiltrate in CRC TME was quantified by the single sample gene set enrichment analysis (ssGSEA) algorithm. 28 immune cell subtypes including MDSC, activated dendritic cells, macrophages, natural killer T cells, and regulatory T cells. The relative abundance value of each TME-infiltrating cell in the sample was expressed by the enrichment fraction calculated by ssGSEA analysis. Patient response to immune checkpoint blockade (CTLA4 and PD1 treatment) was predicted by the Tumor Immune Dysfunction and Rejection (TIDE) tool (http://tide.dfci.harvard.edu/login/). data for TMB were obtained from the TCGA database. MSI scores were obtained from the TIDE database. Immune function pathways were obtained from the ImmPort database (https://www.immport.org/shared/home).

### Genomic mutation analysis

Somatic mutation data were obtained from the TCGA database. Genes with significant mutations in the somatic mutation database were identified by the R package "maftools". The mutation characteristics of the obtained CRC patients were extracted and compared with the mutation database (COSMIC V2), using the cosine similarity method (https://cancer.sanger.ac.uk/cosmic/).

### Cell culture

Procell (Wuhan, China) supplied the human normal colorectal epithelial cell line NCM460 and the CRC cell lines SW620, HCT116, SW480, DLD1, Lovo, and HT29. The cells were cultured in DMEM containing 10% fetal bovine serum at 37 degrees Celsius and 5% carbon dioxide. siRNA targeting TNFRSF10A-AS1 (si- TNFRSF10A-AS1) and the negative control (si-NC) were designed and synthesized by Gene Pharma Technology (Shanghai, China). SW480 and SW620 cells were seeded in antibiotic-free DMEM in a 6-well plate for 24 h, at 70–80% confluence, before being transfected with 50 nM of si-TNFRSF10A-AS1 and 50 nM of si-NC with the aid of Lipofectamine 2000 (11,668,030, Thermo Fisher). The cells were collected after 48 h of transfection.

### CCK-8 Assay

The transfected SW480 and SW620 cells were plated in 96-well plates with a 100 μL cell suspension (1500 cells). CCK-8 (10 μL per well) was then added at a later time and cultured for 2 h. We used a microplate reader (Thermo Fisher) for the purpose of measuring the optical density (OD) at 450 nm.

### Scratch wound assay

SW480 and SW620 cells were planted at a density of cells per well in 6-well culture plates. The cells were cultured in 2 mL of growth media to reach 90% confluence. Subsequently, a 10 μL pipette tip was used to scrape the cell layer. The cells were then treated with allicin after rinsing. We observed the cell cultures immediately, 12, and 24 h later. The progression of cell migration was monitored under a microscope.

### EDU assay

A 24-well plate was used for the seeding of SW480 and SW620 cells. The EdU kit's instructions were followed to make a 2 × EdU reaction solution, which was then added to the 24-well plate. The cells were incubated free of light after being treated with 4% paraformaldehyde at normal room temperature for a period of 20 min, supplemented with 0.3% Triton × -100, and reacted at room temperature for 10 min. Subsequently, PBS was employed in washing the cells three times. 200 μL of newly produced AZIDE 555-Click reaction solution was emptied into each well, and an incubation of the plates was done for half an hour at room temperature and in the dark. After the reaction was done, the solution was discarded, and the cells were then rinsed with PBS thrice before being counterstained with Hoechst for immunofluorescence. They were then observed and captured under an inverted microscope. The inverted microscope was used for the examination and photography.

### qPCR analysis and RNA extraction

RNA was isolated from the cells with the aid of the Hipure Total RNA Mini Kit, (R4111-03, Magen, China). HiScript II QRT SuperMix (Vazyme, China) was then used in conducting the reverse transcription. The SYBR GREEN MIX (Vazyme, China) and the CFX96 Real-time PCR Detection System (Bio-Rad, USA) were employed in the qRT-PCR. GAPDH was selected as the internal control, and we used the 2^−ΔΔCt^ technique to determine relative expression. Each qRT-PCR was conducted thrice.

### Statistical analysis

R software and Perl were employed in statistical analysis. Random sequence was generated by SPSS 22.0. Survival curves were evaluated using the log-rank test and the Kaplan–Meier analysis. ROC curve analysis was employed in the determination of the prognostic signature's predictive performance, with AUC values of 0.6–0.7, 0.7–0.9, and 0.9–1.0 indicating acceptable, moderate, and high performance, respectively. The univariate Cox regression analysis was conducted in the screening of prognostic-related factors, whereas the multivariate Cox regression analysis was conducted in the identification of prognostic-related factors. *p* < 0.05 were considered statistically different.

## Results

### Differentially expressed m5C regulators in colorectal cancer and normal tissue

**Fig. 1:**
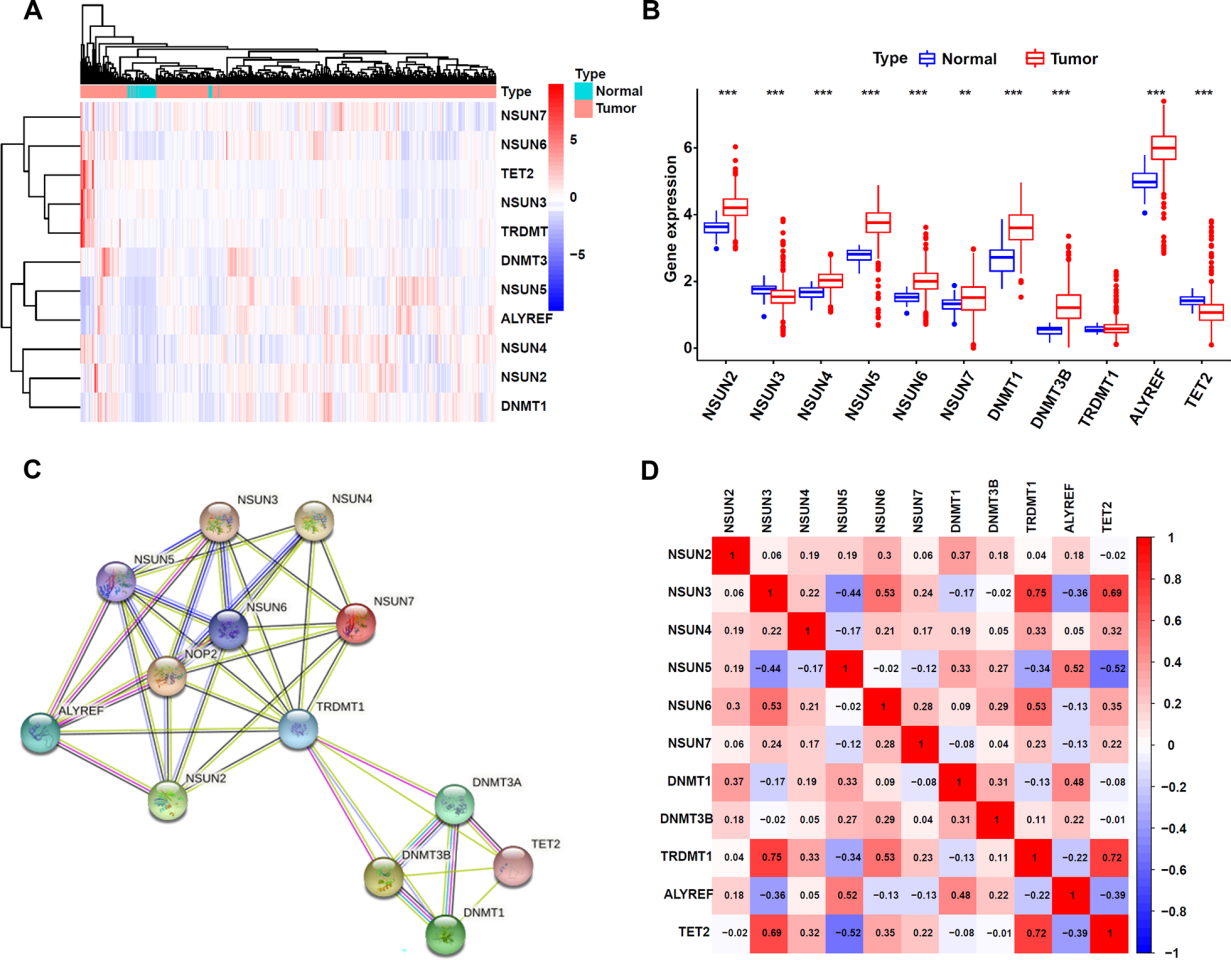
Differentially expressed m5C regulators between breast cancer tissues and non-tumor normal tissues: **A** Heatmap of m5C regulators. The depth of blue indicates the level of low expression, and the depth of red indicates the level of high expression. **B** Violin plot for the m5C regulators, the blue column indicates normal tissue and the red column indicates tumor tissue. **C** Protein–Protein Interaction (PPI) network analysis of m5C regulators in CRC. **D** Co-expression analysis for m5C regulators, the red color indicates a positive correlation and the blue color indicates a negative correlation. *p < 0.5, **p < 0.01, and ***p < 0.001. *ns* no sense

We examined 548 patients suffering from CRC and 44 controls using the TCGA database to determine the differentially expressed genes as well as the roles they played in m5C regulation in CRC. The TCGA-READ and TCGA-COAD datasets' pertinent RNA-seq and clinical data, comprising 548 CRC cancer tissues and 44 healthy adjacent tissues were acquired from TCGA's database (Table 1). Figure 1A displays heatmaps of m5C regulators, whereas Fig. 1B displays the expression of m5C regulators as shown in the form of Violin Plot. NSUN2, NSUN5, NSUN4, NSUN7, NSUN6, DNMT3B, DNMT1, and ALYREF (*p* < 0.05) all had remarkably up-regulated expression, whereas NSUN3 and TET2 both had a significantly down-regulated expression (*p* < 0.05). The STRING database was then searched to develop a protein–protein correlation network (Fig. 1C). The PPI network node TRDMT1 has the most links to other genes (12 in total). Co-expression analysis and the Pearson correlation coefficient both confirmed this association (Fig. 1D). The results showed that TRDMT1 also has the strongest association with other genes, NSUN3, NSUN5, NSUN6, ALYREF, and TET2 has a strong association with other genes correlated (correlation coefficient ≥ 0.5). Based on these findings, there is a strong link between m5C regulators and CRC.

### m5C-related lncRNAs’ identification and the prognostic signature model's development

**Fig. 2:**
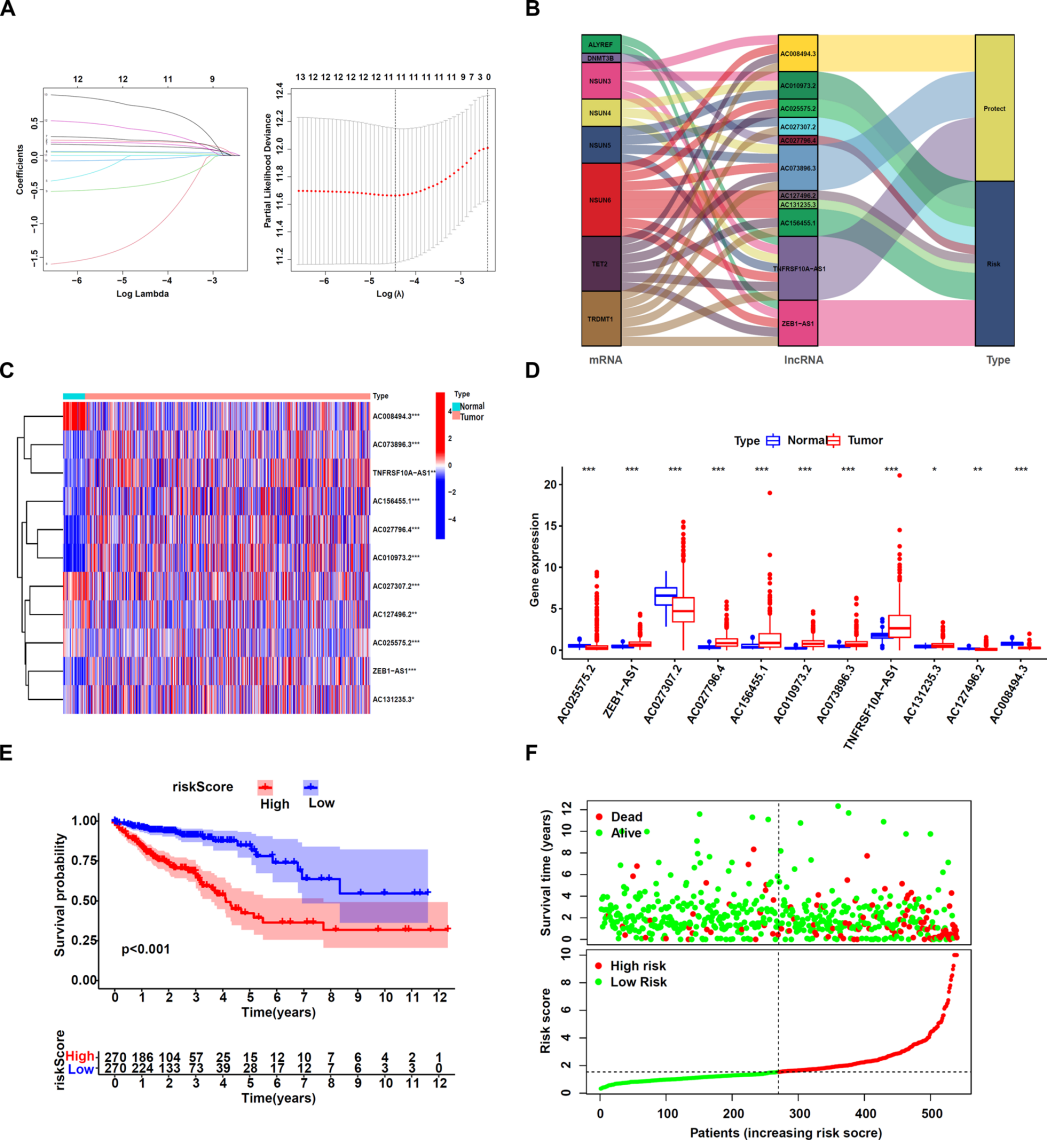
Key prognostic-related LncRNAs and construction of prognostic risk signature: **A** LASSO Cox regression of 11 lncRNAs used in the prognostic risk model and LASSO filters variables **B** The Sankey plot demonstrated the relationship between the m5C regulators, m5C-related prognostic signature lncRNAs. **C** Heatmap of the m5C-related prognostic signature lncRNAs. The depth of blue indicates the level of low expression, and the depth of red indicates the level of high expression. **D** Vioplot for the m5C-related prognostic signature lncRNAs, the blue column indicates normal tissue and the red column indicates tumor tissue. **E** KM curve shows that patients in the m5C-related lncRNA low-risk group survived dramatically longer than those in the high-risk group. **F** The scatter plot displayed the risk score distribution of high-risk and low-risk CRC patients based on the m5C-related lncRNA risk model and the relationship between survival time and CRC patients' risk score. *p < 0.5, **p < 0.01, and ***p < 0.001. *ns* no sense

Pearson correlation analysis was conducted to identify the m5C-related lncRNAs based on the expression of m5C regulators and lncRNAs in CRC patients. We could define a lncRNA as the m5C-associated lncRNA if its expression was significantly associated with one or more m5C regulators (*p* < 0.001 and |correlation coefficient|> 0.3) acquiring 574 m5C-related lncRNAs. We conducted the univariate cox regression analysis to isolate m5C-associated lncRNAs that were significantly linked to prognosis (*p* < 0.05). As per the aforementioned criteria, 13 lncRNAs were tested for their role in CRC prognosis; the majority of the m5C-associated lncRNAs were significant risk factors for CRC (HR > 1), whereas AC073896.3, AC008494.3, and TNFRSF10A-AS1 were protective factors (HR < 1) (Additional file1: Fig S1). The findings of an initial screen of these lncRNAs using the LASSO regression method indicated that these 11 m5C-related lncRNAs were appropriate for developing the prognostic risk model (Fig. 2A). This is how we determined the risk score: = 0.142973474053793* AC025575.2 + 0.224469313148377* ZEB1-AS1 + 0.0638241231289148* AC027307.2 + 0.0242317002865264*AC027796.4 + 0.203995411959886*AC156455.1 + 0.24375190773417*AC010973.2–1.15592494503022*AC008494.3–0.22746636611653*AC073896.3–0.0991526739877792*TNFRSF10A-AS1 + 0.136023395284555* AC131235.3 + 0.704950993998284* AC127496.2. This association between m5C-associated prognostic signature lncRNAs, m5C regulators, and the effect of these lncRNAs on prognosis was graphically shown using a Sankey plot (Fig. 2B). Finally, the TCGA database and the GEO database were applied in an attempt to acquire insight into the expression of the screened predicted m5C-associated lncRNAs in patients suffering from CRC. All of the lncRNAs showed statistically significant variations between normal and tumor colorectal tissues as shown by the heatmap and the Table 2, suggesting that the m5C-associated lncRNAs may partake a critical part in CRC development (Fig. 2C, D, Additional file2: Fig S2).

**Table 2:** Expression of m5C-related lncRNAs in the GEO database

Gene	Analysis Id	n tumor *vs*. normal	logFc	Average expression	*p*-value	Adjusted *p*-value
AC027307.2	GSE21510	148 (123–25)	− 0.7612	6.1872	0	0
	GSE18105	111 (94–17)	− 0.3347	6.0338	0	0.0002
	GSE39582	585 (566–19)	− 0.335	5.4259	0.0001	0.0015
	GSE37364	65 (27–38)	− 0.201	7.4719	0.01	0.0332
AC027796.4	GSE90524	6(3–3)	− 1.6866	7.7629	0.0007	0.0157
ZEB1-AS1	GSE21510	148 (123–25)	0.869	5.3455	0	0
	GSE18105	111 (94–17)	0.8987	5.3567	0	0.0001
	GSE37364	65 (27–38)	0.5233	5.1887	0	0.0002
	GSE39582	585 (566–19)	0.5283	4.0832	0.0003	0.0029
	GSE83889	136 (101–35)	0.2574	4.8208	0.004	0.0177
	GSE9348	82 (70–12)	0.432	3.8851	0.0033	0.0236
AC010973.2	GSE81558	51 (42–9)	0.5744	6.3700	0.0000	0.0000
TNFRSF10A-AS1	GSE90524	6 (3–3)	− 2.263	2.9529	0	0.0067
AC073896.3	GSE90524	6 (3–3)	− 2.1082	4.6954	0.0013	0.0209
AC008494.3	GSE39582	585 (566–19)	− 0.2915	2.7917	0	0
	GSE5206	105(100–5)	− 0.3807	4.6154	0	0
	GSE21510	148 (123–25)	− 0.367	3.5455	0	0
	GSE18105	111 (94–17)	− 0.245	3.4663	0	0
	GSE37364	65 (27–38)	− 0.4594	4.2469	0	0
	GSE9348	82(70–12)	− 0.2625	3.0823	0	0
	GSE50421	49 (24–25)	− 0.2832	6.5339	0.0027	0.0243

We used the calculated risk score method and the median risk value to group the 540 patients suffering from CRC into high-risk and low-risk groups. Kaplan–Meier analysis of survival data (Fig. 2E) demonstrated that OS (overall survival) was higher for patients in the low-risk group compared to those in the high-risk group (*p* < 0.001). The scatter plot and risk curve depicted the correlation between the corresponding survival status in CRC victims and the risk score (Fig. 2F), with a greater risk score being linked to a higher rate of mortality. Therefore, using a panel of 11 m5C-associated lncRNAs, we were able to determine the prognosis significance and identify m5C-associated lncRNAs with substantial prognostic significance.

### Relationship between clinicopathological variables and m5C-related lncRNAs’ differential expression

**Fig. 3:**
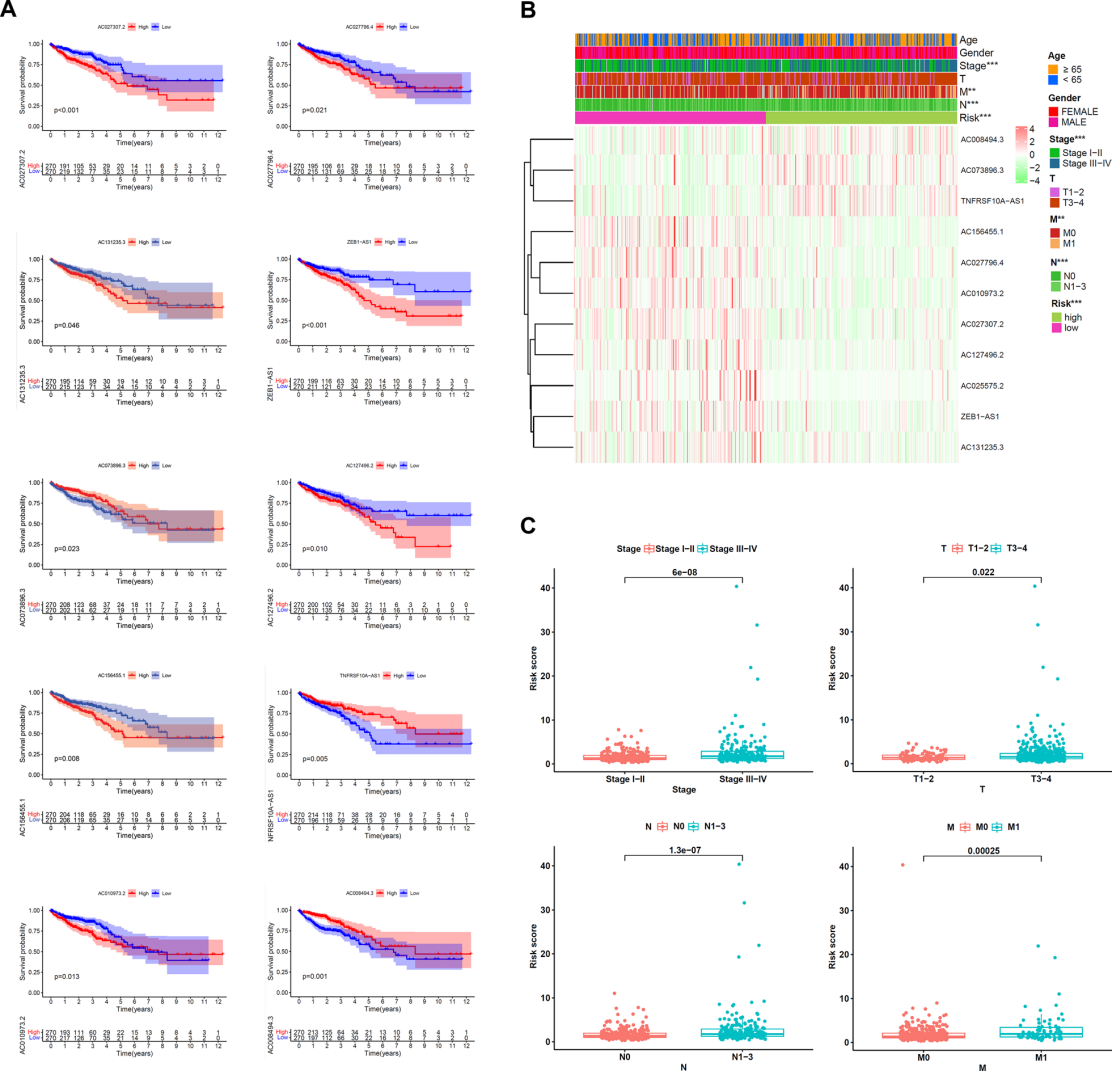
Relationships between m5C-related lncRNAs and clinical pathological parameters. **A** Ten survival curves based on the m5C-related lncRNAs expression. **B** Heatmap displayed the clinical characteristics and differences in the high- and low-risk group calculated by m5C-related lncRNA risk scores. **C** Risk scores in subgroups including stage, T stage, N stage and M stage

We further examined the overall survival time of the selected 11 lncRNAs for patients suffering from CRC. The m5C-associated lncRNAs in the OS curve illustrated that individuals with high-risk lncRNA high expression (AC027307.2, AC027796.4, AC131235.3, ZEB1-AS1, AC127496.2, AC156455.1, AC010973.2, AC008494.3) had a shorter survival timeframe, while those patients with protective lncRNA high expression (AC073896.3, TNFRSF10A-AS1) had a longer survival timeframe (Fig. 3A). The pathological stage (*p* < 0.001), N stage (*p* < 0.001), and M stage (*p* < 0.01) depicted statistically significant variations between the high-risk and low-risk groups, as shown by the heatmap. No remarkable differences in terms of gender, age, or T stage (Fig. 3B) were recorded. We then categorized these clinical markers into subgroups and evaluated their risk score values. The expression of risk scores showed that risk scores in stage III–IV group, T III–IV group, N I–III group, and MI group were higher than in stage I–II group, T I–II group, N0 group, and M0 group (Fig. 3C). According to the KM survival curve, subjects with high-risk ratings had a shorter OS in the following subgroups: patients not older than 65 years, male subjects, Stage I–II patients, T I–II or T III–IV patients, N0 or N1–III patients, and subjects without any metastases group (Additional file3: Fig S3).

### Prognostic model verification and nomogram construction.

**Fig. 4:**
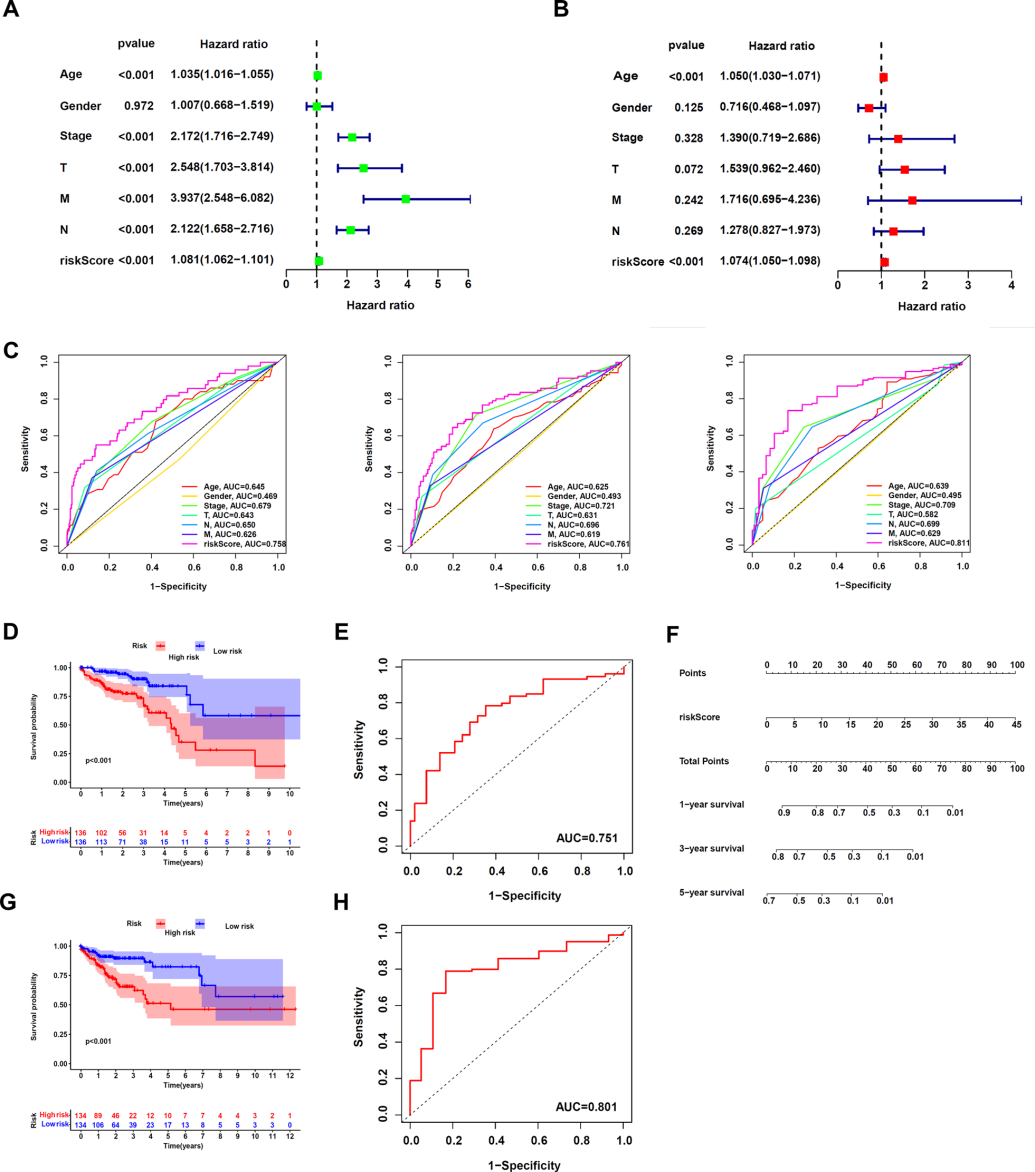
Verification of the risk model and construction of the nomogram. **A**, **B** Univariate and multivariate Cox regression analysis of the prognostic value of risk scores and clinical features. **C** Determination of the area under the ROC curve (AUC) of the risk score and clinical characteristics based on the ROC curve. **D**, **E**, **G**, **H** Overall survival and ROC analysis in subgroups (D, E: Group A; G, H: Group B). **F** A nomogram model was established using risk score

We performed multivariate and univariate Cox regression analysis to establish whether risk scores could be used as an independent prognostic factor. We found that the HR for risk score = 1.081, 95% CI 1.062–1.101 (*p* < 0.001) in univariate Cox regression, and in multivariate Cox regression, the HR for risk score = 1.074, 95% CI 1.050–1.098 (*p* < 0.001). This showed that risk score is a crucial prognostic factor that can be independent of sex, pathological stage, age, and TNM stage (Fig. 4A, B). For the purpose of determining its accuracy in prognosis prediction, we calculated the area under the receiver operating characteristic (AUC) curve for CRC patients' risk assessment scores. The 1-year, 3-year, and 5-year AUC values we discovered for the risk score were 0.758, 0.761, and 0.811, respectively; these values were higher than those identified for other clinicopathological characteristics (Fig. 4C). These results suggested that m5C-associated lncRNAs is significantly independent of prognostic factors in patients suffering from CRC. The prognostic model's accuracy in predicting 1-, 3-, and 5-year overall survival for CRC patients was demonstrated in time-dependent ROC analysis (Additional file4: Fig S4). Additionally, we did an internal validation of the m5C-associated lncRNA risk model by randomly splitting all of the TCGA. CRC subjects into two subdivisions (group B and A) at a ratio of 1:1. For each group, researchers looked at the KM survival curve and the 5-year ROC curve. Results from our study indicated that patients in group A with greater m5C-connected lncRNA risk scores had shortened overall survival duration (HR: 2.05, 95% CI 1.13–3.71, *p* = 0.019) and that the AUC value of the 5-year ROC curve was 0.751 (Fig. 4D, E). Subjects in Group B also exhibited a decreasing trend in OS (HR: 2.56, 95% CI 1.44–4.56, *p* = 0.001), and their AUC value was 0.801 (Fig. 4G, H). Concurrently, we developed a nomogram using the risk score as a quantitative tool for predicting patients' outcomes in clinical settings (Fig. 4F). Based on these findings, it is clear that the m5C-connected lncRNA risk model is a robust predictive factor of CRC.

### Variations of the m5C status of low-risk and high-risk groups

**Fig. 5:**
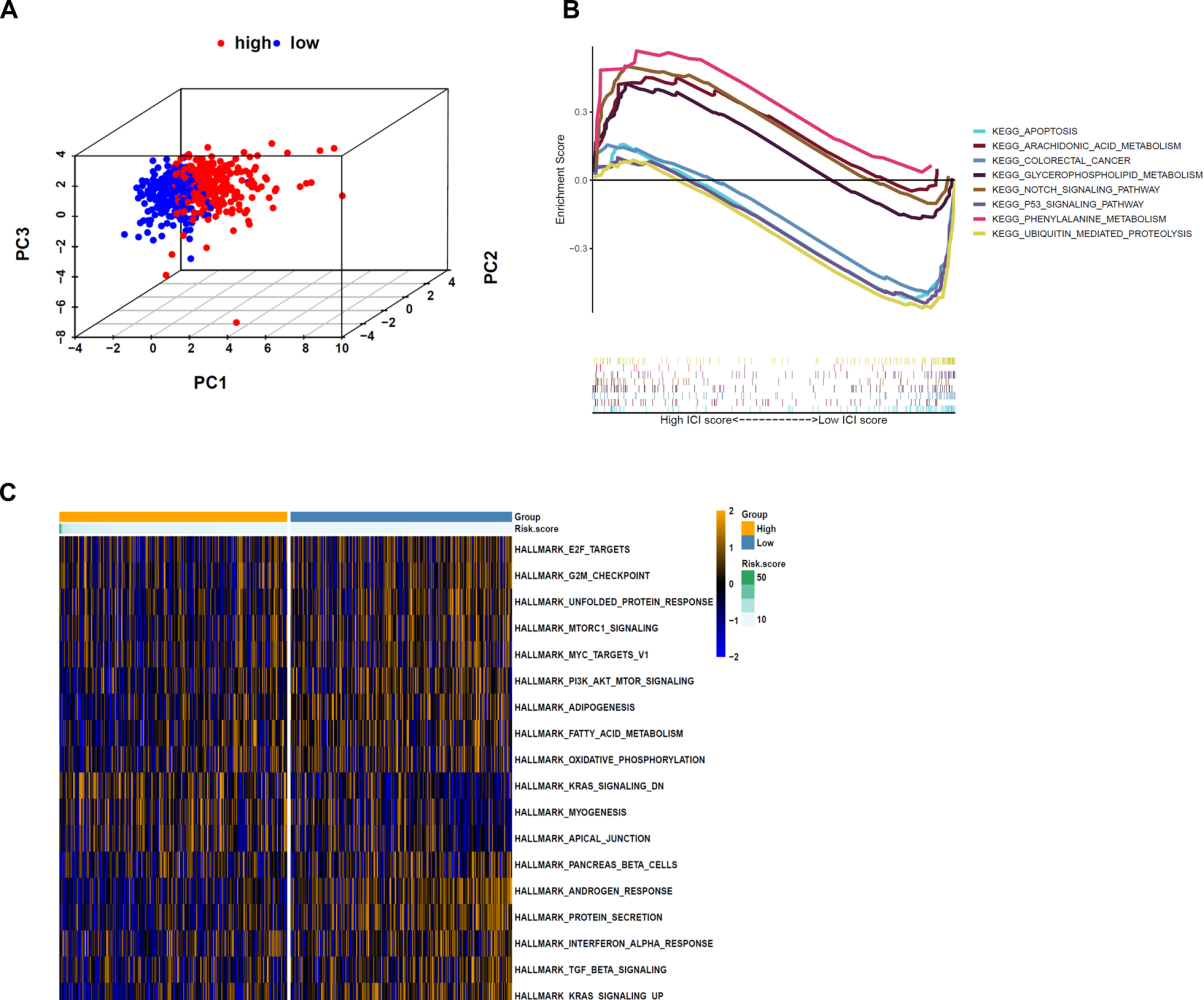
The m5C status was different between the high- and low-risk groups and functional annotation of the two risk groups. **A** Principal component analysis (PCA) was performed for the low- and high-risk groups based on the whole genome and m5C-related coding genes, and a risk model was constructed using 11 m5C-related lncRNAs. **B** Top enriched gene pathways in low- and high-risk groups from the CRC cohort were assessed by using the GSEA algorithm. **C** GSVA enrichment analysis between the low- and high-risk clusters, and yellow represents activated and blue represents repressive pathways

Principal component analysis was conducted in classifying patients into high-risk and low-risk groups (categories) as per their expression levels of lncRNAs associated with m5C (Fig. 5A). The distribution of subjects into high-risk and low-risk groups, as determined by m5C-associated lncRNAs, is clear. Therefore, the prediction model's specificity and sensitivity were shown by the fact that m5C-associated lncRNAs could classify patients with CRC into two categories. GSEA was carried out to establish the potential signaling pathways engaged by lncRNAs associated with m5C in the low- and high-risk cohorts. High-risk individuals showed upregulation of pathways involved in phenylalanine metabolism, notch signaling, and arachidonic acid metabolism, whereas low-risk individuals showed upregulation of COLORECTAL CANCER and P53 SIGNALING PATHWAY (Fig. 5B). GSVA enrichment analysis showed that m5C-associated lncRNAs were significantly associated with pathways related to tumor progression, such as KRAS signaling pathway, PI3K-AKT-mTOR signaling pathway, MYC targets and TGF-BETA signaling pathway, and so on (Fig. 5C).

### Evaluation of the association between models of immune cell infiltration and m5C-related prognostic signature

**Fig. 6:**
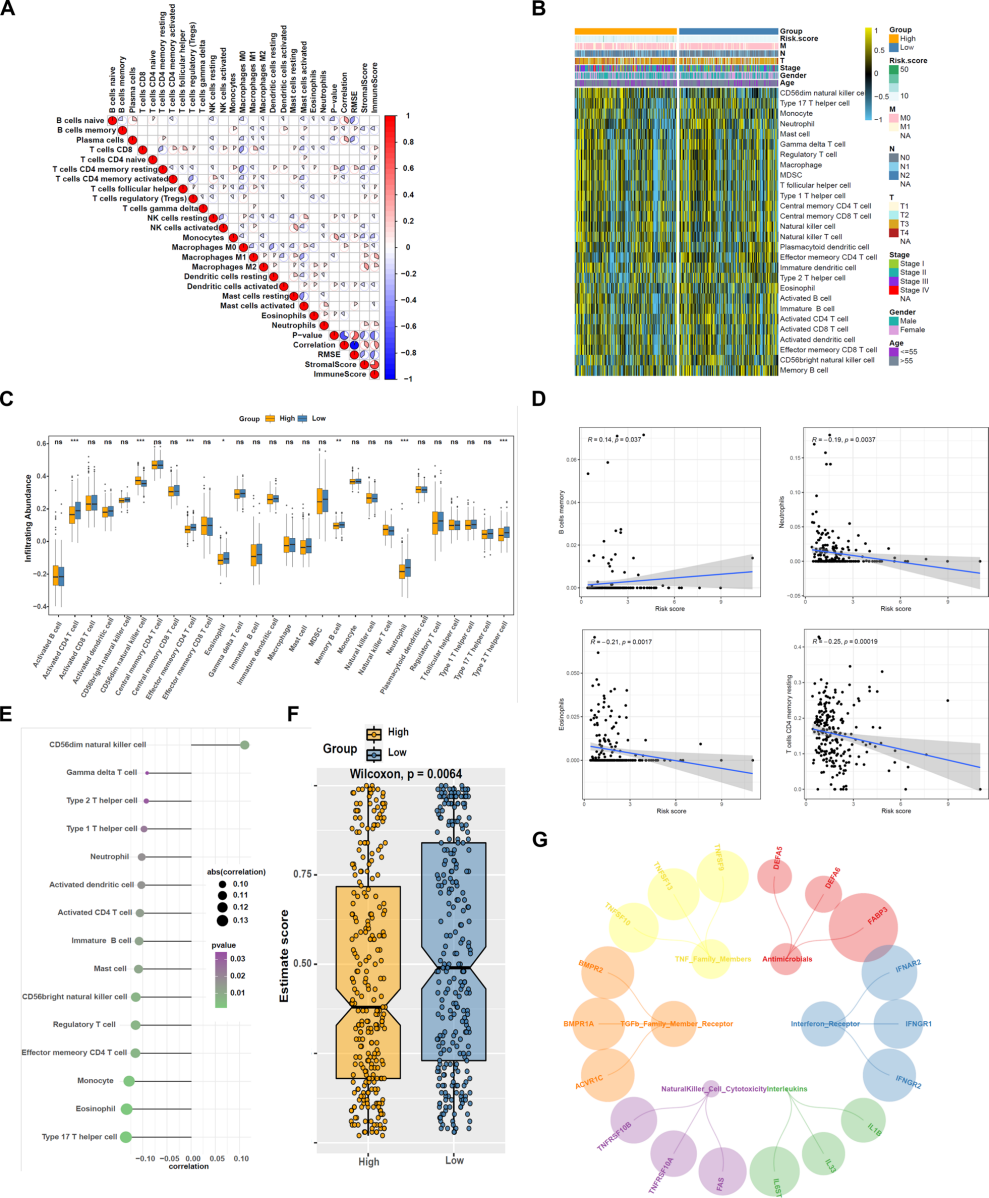
Correlation between tumor-infiltrating immune cells and risk model. (**A**) Spearman correlation analysis of 28 tumor-infiltrating immune cells. **B**, **C** Heatmap and violin plot of 28 tumor-infiltrating immune cell types in low- and high-risk groups. **D** Correlation of risk score with 4 tumor-infiltrating immune cell subtypes. **E** The correlation between risk scores and immune infiltration cells in TCGA melanoma. **F** MSI score analyses among the high- and the low-risk groups. **G** Six immune-related pathways expressed between the high- and the low-risk groups

We evaluated relationships between immune/stromal/ESTIMATE scores and immune cells, a positive correlation (red circle) and a negative correlation (blue circle) were established (Fig. 6A). We compared the proportion of 28 distinct TIIC across low-risk and high-risk patients suffering from CRC using the CIBERSORT approach and presented our findings using heatmaps, violin plots and bubble chart (Fig. 6B, C, E). There was a statistically significant (*p* < 0.05) increase in the numbers of CD56dim natural killer cell in the high-risk groups, whereas the numbers of Activated CD4 T cell, Effector memory CD4 T cell, Eosinophil, Memory B cell, Neutrophil and Type 2 T helper cell decreased (*p* < 0.05). Both eosinophil and T cells CD4 memory resting were highly inversely linked to risk score, whereas risk score was strongly positively linked to B cells memory (Fig. 6D). MSI score analyses showed that different response of immunotherapy among the high- and the low-risk score groups (Fig. 6F). Therefore, we hypothesized that the variability in immunotherapy that appears in different subgroups of CRC patients may be due to several specific pathways. We then identified six immune-related pathways with differential expression in the high- and low-risk groups (*p* < 0.05) (Fig. 6G). Based on these results, it seems that the risk profile of m5C-associated lncRNAs can discriminate between tumor immune cells’ characteristics in CRC.

### Analysis of mutation patterns and chemotherapy between high- and low- risk groups

**Fig. 7:**
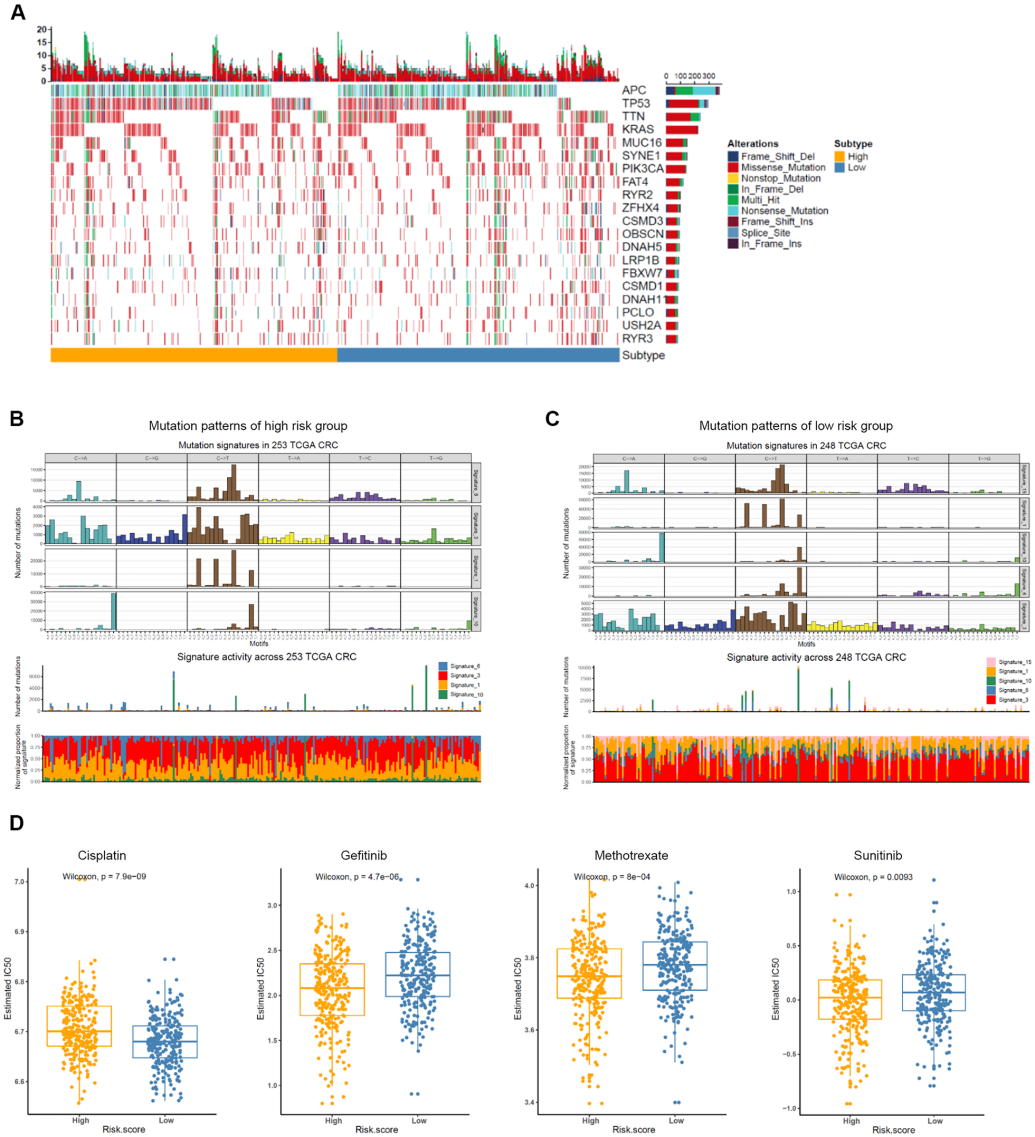
Comparison of mutational patterns and signatures in the two risk subtypes of TCGA CRC samples. **A** The waterfall plot of tumor somatic mutation was established by those with high-risk and low-risk. **B**, **C** Mutation signature extracted in the high risk group and low risk group. **D** The IC50s of chemotherapeutic agents with ferroptosis score, listed by cisplatin, gefitinib, methotrexate and sunitinib

We analyzed CRC samples and SMG mutation profiles to explore the association between immune cell infiltration and mutation patterns. The results showed a significant proportion of mutations in TP53, TTN and KRAS, and so on (Fig. 7A). We performed SMC and extracted mutation signatures from the COSMIC database by using genomic somatic mutation data from CRC for analysis to understand mutation signatures among immune infiltrating cells in each subgroup (Fig. 7B, C). The result revealed that low-risk group had the independent characteristics of signature15. These results suggested that the mutation pattern in low-risk group was associated with defective DNA mismatch repair. In addition, we performed a predictive analysis of chemotherapy response in both groups by applying the pRRophetic R package. Patients in the low-risk group had a higher sensitivity to the following chemotherapeutic agents: Cisplatin (Wilcoxon rank sum test, *p* = 7.9e–09). Patients in the high-risk group had a higher sensitivity to Gefitinib (Wilcoxon rank sum test, *p* = 4.7e–06), Methotrexate (Wilcoxon rank sum test, *p* = 8e–04), Sunitinib (Wilcoxon rank sum test, *p* = 0.0093) (Fig. 7D).

### Verification of m5C-related lncRNA TNFRSF10A-AS1 in vitro in the CRC cells

**Fig. 8:**
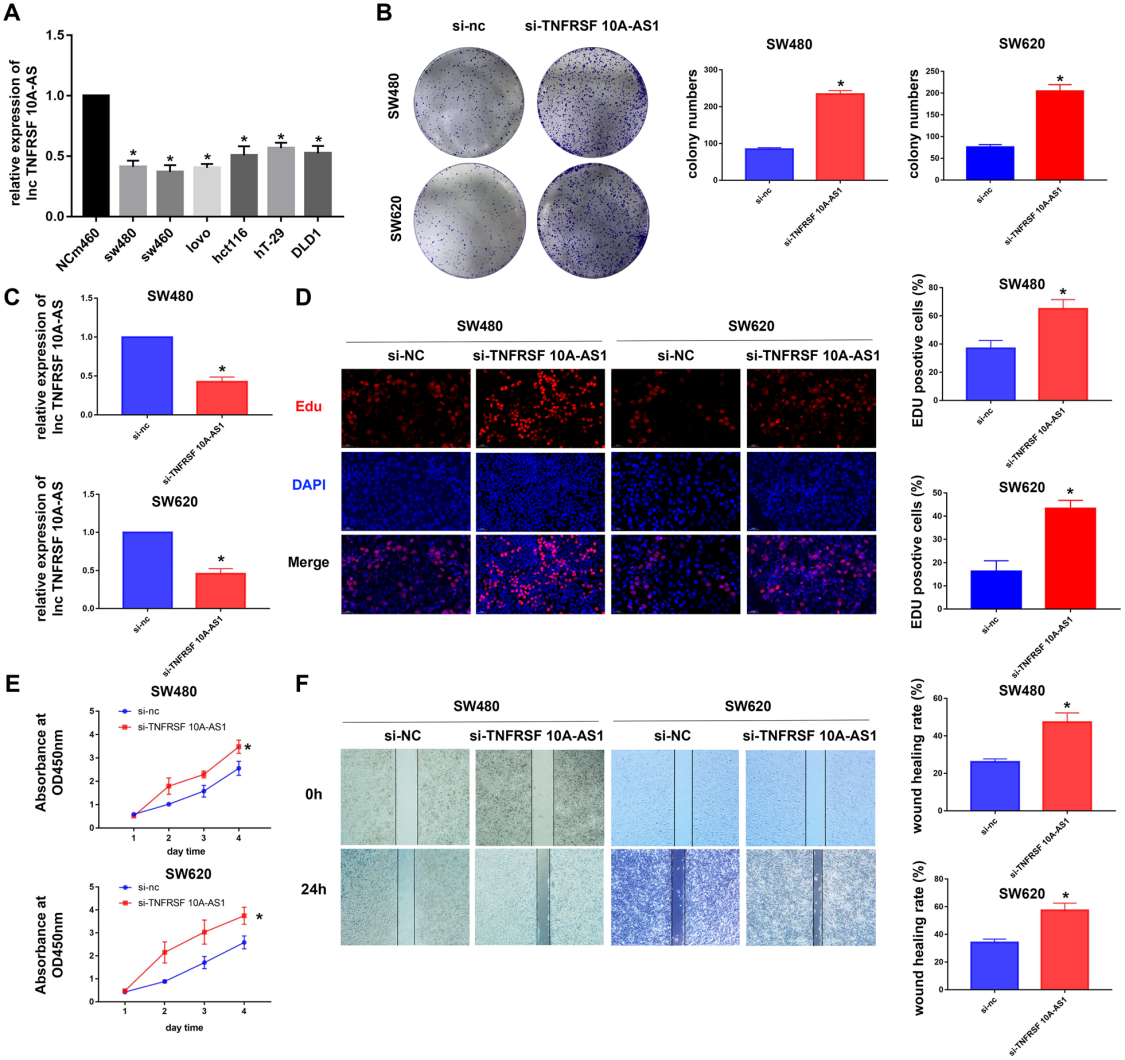
Verification of m5C-related lncRNA TNFRSF 10A-AS1 in the CRC cells. **A** qPCR was performed to detect the expression of TNFRSF 10A-AS1 in all types of CRC cells. **B** The effect of TNFRSF 10A-AS1 on cell proliferation was determined by the clone formation assay. **C** qPCR was performed to detect the expression of TNFRSF 10A-AS1 in transfected si-NC and si-TNFRSF 10A-AS1 colorectal cancer cells. **D** The effect of TNFRSF 10A-AS1 on cell proliferation was measured by the Edu assay. **E** The effect of TNFRSF 10A-AS1 on cell viability was determined by the CCK-8 assay. **F** A wound-healing test was performed to assess the effect of TNFRSF 10A-AS1 on cell migration. *p < 0.5, **p < 0.01, and ***p < 0.001. ns, no sense

TNFRSF10A-AS1 was chosen for the in vitro verification of the m5C-connected lncRNA to explore the effect of m5C-related lncRNA in the colorectal cell, SW480, and SW620 colorectal cancer cells were split into two groups: the NC and si-TNFRSF10A-AS1 groups, respectively. Q-PCR results showed that the expression level of TNFRSF 10A-AS1 in Human normal colorectal epithelial cell line NCM460 was higher than its expression in colorectal cancer cell line SW480, SW620, DLD1, HCT116, HT29, and Lovo cells (Fig. 8A). The results recorded from the wound-healing test, CCK-8 assay, clone formation assay, and Edu assay results showed that TNFRSF 10A-AS1 significantly decreased cell proliferation (Fig. 8B, D, E). 24-h wound-healing test showed that decrease of TNFRSF10A-AS1 in CRC cells weakened their migration capability (Fig. 8F). TNFRSF10A-AS1 demonstrated impressive effects on the proliferation, migration and invasion of CRC cells.

## Discussion

With the improvement in people's living standards, colorectal cancer has increasingly become one of the cancers threatening human health. Although aggressive multimodal treatment regimens (chemotherapy, surgery, targeted therapy, radiotherapy, and immunotherapy) have immensely boosted the survival of patients suffering from CRC, treatment outcomes are still unsatisfactory. As a result, it is of significant value to look for new and operative therapeutic targets for the diagnosis and management of CRC.

Epigenetics is the study of heritable and reversible phenotypes such as DNA and RNA methylation, noncoding RNA modifications, histone modifications, and chromatin rearrangements. More than a hundred distinct RNA chemical modifications have been identified, making the study of epigenetics a rapidly growing field [38–40]. Methylation of RNA is a universal post-transcriptional alteration that plays a crucial role in the regulation of several biological processes, such as splicing, transcription, stability, structure, and translation. Human cancers are linked to their dysregulation [41]. Gene expression and disease progression are regulated by RNA post-transcriptional modifications, the most prevalent of which are m6a, m5C, and m1A [17], 42, 43. It has been shown, however, that aberrant RNA alterations lead to a number of illnesses, including cancer [44, 45]. For this study, we used the TCGA database to retrieve the gene expression profiles of 540 CRC patients and developed a risk model based on lncRNAs linked to the 11-methylcytosine (m5C) mutation. We believe this is the first study to examine the predictive value of lncRNAs linked to the m5C regulator in colorectal cancer.

Gene expression may be regulated by lncRNA in several ways, including transcriptional regulation, mRNA stability, and translational control [46, 47]. In addition, lncRNA can act as guides, scaffolds, or decoy molecules for proteins to recruit proteins or RNA. lncRNAs can also affect the structure of chromatin and lead to the regulation of gene expression [48]. We employed bioinformatics and statistical methodologies to develop a CRC predictive risk model, with a special emphasis on lncRNAs that have co-expression links with m5C regulators in CRC.

In our study, 13,142 m5C-connected lncRNAs were recognized from the TCGA-CRC dataset to probe its prognostic value for patients suffering from CRC. Importantly, we used the m5C-connected lncRNAs to develop a prognostic signature by LASSO Cox regression analysis to forecast the prognosis of patients with CRC. The prognostic signature was shown to be effective in classifying CRC patients into high-risk and low-risk categories by means of time-dependent ROC analysis, Kaplan–Meier analysis, multivariate, and univariate Cox regression analysis. It can be used as an independent factor for CRC patients’ outcomes. Furthermore, a nomogram was used to validate this prognostic signature, which would be easier for the clinician to use our model in daily clinical work. Among these signature, TNFRSF10A-AS1 was reported as an autophagy-related long noncoding RNA in colorectal cancer patients by bioinformatic analysis [49]. An independent prognostic factor, TNFRSF10A-AS1, played a crucial oncogenic function in GC. It was shown that MPZL1 was a direct downstream effector of TNFRSF10A-AS1 that was necessary for its oncogenic action [50]. As TNFRSF10A-AS1 promotes tumor onset and progression in CRC via the miR-3121-3p/HuR axis, it has the potential to be a new therapeutic target for this disease [51]. Additionally, the remaining lncRNAs were rarely reported by other authors.

We compared the immune infiltration in low- and high-risk CRC patients and found that NK cells were significantly upregulated in the high-risk group. Although it has been reported that CD8 + T cells, M1 and M2 TAMs are identified to play an important role in the development and progression of CRC, however, regarding the high density infiltration of NK cells in the tumor microenvironment is also important to inhibit tumor growth and metastasis in solid tumors such as colorectal cancer, lung cancer, etc. Its role in immunotherapy is also gradually recognized. In addition, the results of tumor immune dysfunction and rejection (TIDE) validate the MSI analysis, with a higher response to immunotherapy in the low-risk group of CRC patients. The above also suggests that our m5C-regulated related lncRNAs may also be involved in immunoregulatory processes in the tumor microenvironment.

Mutation analysis revealed that the top three in both risk groups were TP53, TTN and KRAS. Tumor suppressor gene TP53 is the most commonly mutated gene in cancer [52]. Mutations in TP53 regulate the ability of p53 to promote apoptosis and ferritin bodies and are involved in the progression of a variety of tumors including colorectal cancer [53]. Mutations in the KRAS gene are associated with CRC onset, progression and mutations in KRAS gene are inextricably linked to the development, progression and prognosis of CRC, as well as to its drug and radiation therapy [54]. As for TTN, it is still little studied in colorectal cancer research. Colorectal cancer is the third most occurring cancer in the world. Drug therapy regarding colorectal cancer has been the focus of attention. In this study, we found that the drug sensitivity of cisplatin for patients in the low-risk group reflected a greater significance. And cisplatin, as a common clinical chemotherapeutic agent, is the most effective chemotherapeutic agent for the treatment of colorectal cancer, together with fluorouracil (5-Fu) and oxaliplatin [55].

Finally, the GSEA analysis points to several pathways for future research. COLORECTAL_CANCER was exactly the type of cancer we are studying. PHENYLALANINE_METABOLISM has received increasing attention in cancer research. The findings of this research showed phenylalanine levels have substantial potential etiological and diagnostic significance since they demonstrate that alterations in the metabolome and microbiome occur at very initial stages during the onset and progression of colorectal cancer [56]. The tumor-suppressor protein p53 of P53 SIGNALING PATHWAY is known as the guardian of the genome. p53 is involved in the activation of various biological responses, mainly including cell cycle arrest, DNA repair, and apoptosis[57, 58]. Activation of p53 is mediated by multiple stress signals, including hypoxia, DNA damage, and strong proliferative signals [59, 60]. Dysregulation of p53 function can be detected in approximately 90% of cancers, including TP53 mutations or abnormal activation of other upstream factors [61]. The NOTCH_SIGNALING_PATHWAY played a key role in the progression of colorectal cancer (CRC), which may affect overall survival (OS) [62]. NOVA1-mediated SORBS2 promoted the migration of CRC by Activating the Notch Pathway, indicating its potential as a therapeutic target. The cell cycle G2/M phase is lengthened [63] when the 5-methylcytosine writers DNMT2 and TRDMT1 are knocked down in senescent colorectal cancer cells.

Nevertheless, our article was not without limitations. For instance, the data set used in the original research was rather insufficient. All we got from TCGA were survival, clinicopathological characteristic, and follow-up data, as well as data on lncRNA expression level. Additionally, the m5C level of m5C-connected lncRNAs needs to be established by a bunch of investigations, like RIP m5C-RNA-BisSeq and m5C-MeRIP-seq. Moreover, additional animal models and human patients suffering from CRC should be used to confirm the in vitro findings in the future. However, we found that our signature model based on m5C-related lncRNAs had high prediction accuracy and clinical applicability, suggesting that it has the potential to guide personalized treatment.

## Conclusion

**Fig. 9:**
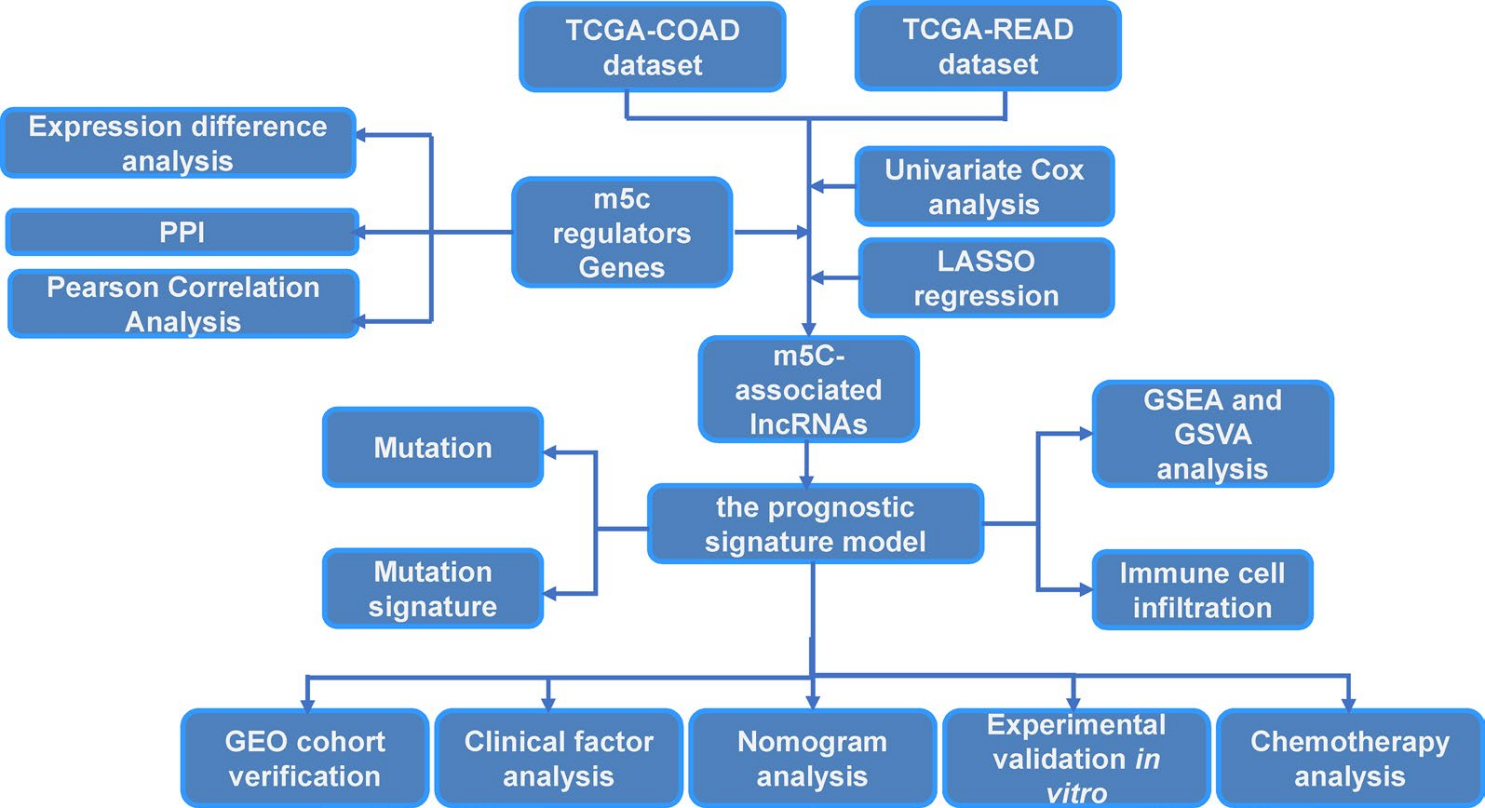
Flow chart of this research

In summary, we analyzed the prognostic significance of m5C-related lncRNAs and developed a nomogram and risk-score signature that can accurately predict the prognosis of CRC patients (Fig. 9). Furthermore, we also revealed the relationship between m5C-related immune cell infiltration and lncRNAs. This study provided some novel insight for m5C-related lncRNAs research, further investigation is needed to elucidate the relevant mechanism.

### Supplementary Information

**Additional file 1: Figure S1**. Forest diagram of univariate Cox Regression analysis of m5C lncRNAs.

**Additional file 2: Figure S2. **The expression of m5C-related lncRNAs in GEO datasets.

**Additional file 3: Figure S3. **Prognostic value of the model based in subgroups including age, gender, stage, T stage, N stage, M stage on Kaplan Meier survival analysis.

**Additional file 4: Figure S4.** The calibration curve of the 1-, 3-, and -5 years overall survival between the actual observation and nomogram prediction.

## Data Availability

RNA-seq data generated in this study are publicly available in TCGA and GEO database. All relevant data can be obtained from the author upon request.
