# Supplementary figures and images for: Identification and validation of a novel prognosis model based on m5C-related long non-coding RNAs in colorectal cancer
Source: Cancer Cell Int. 2023 Sep 5;23:196. doi: 10.1186/s12935-023-03025-2 (PMC10481501; doi:10.1186/s12935-023-03025-2)

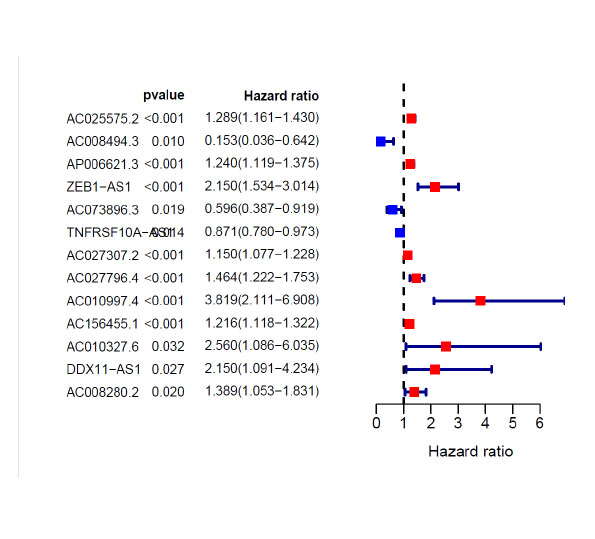

Supplement: Supplementary file 1 — Additional file 1: Figure S1. Forest diagram of univariate Cox Regression analysis of m5C lncRNAs. [file 12935_2023_3025_MOESM1_ESM.jpg]

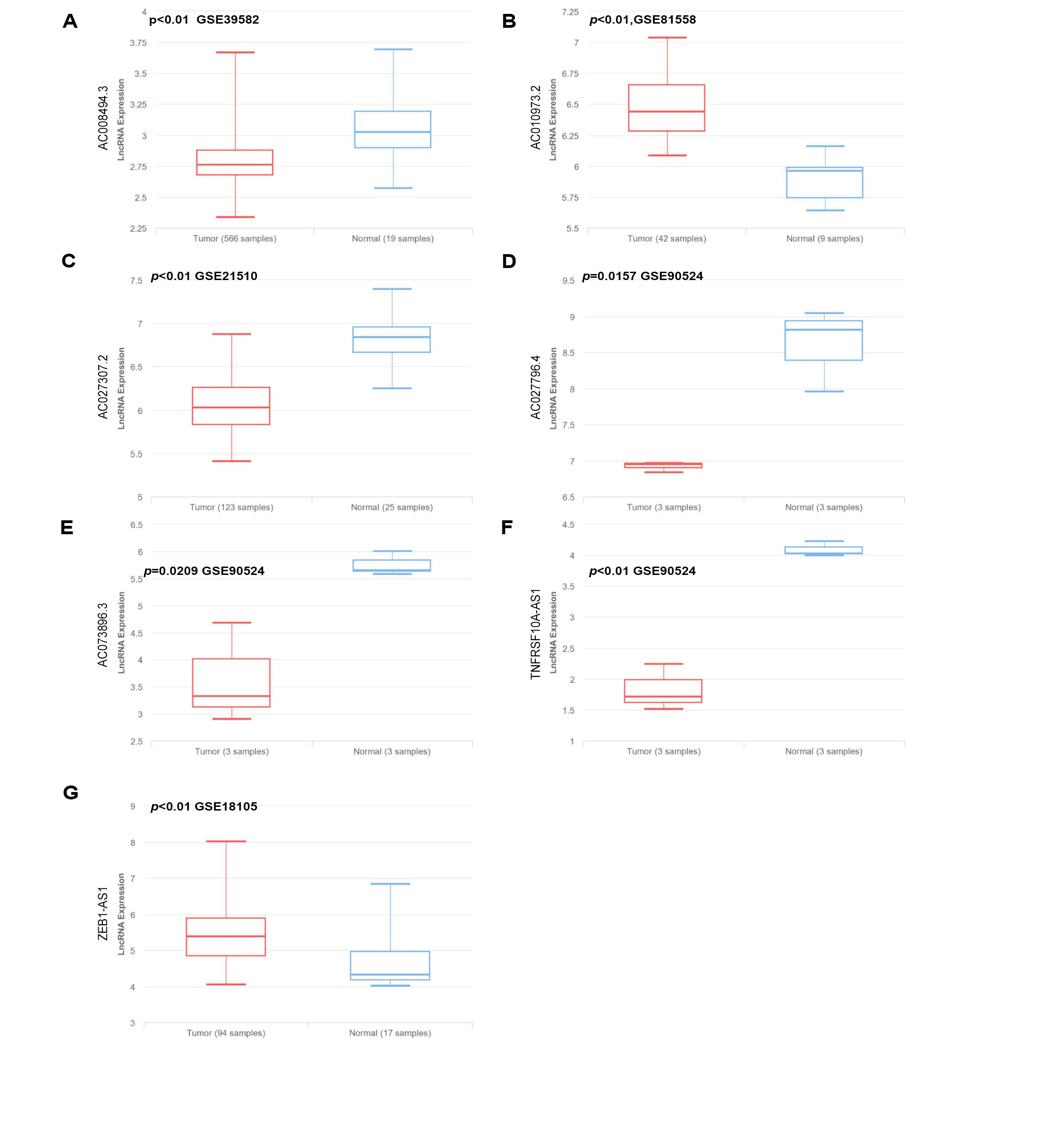

Supplement: Supplementary file 2 — Additional file 2: Figure S2. The expression of m5C-related lncRNAs in GEO datasets. [file 12935_2023_3025_MOESM2_ESM.jpg]

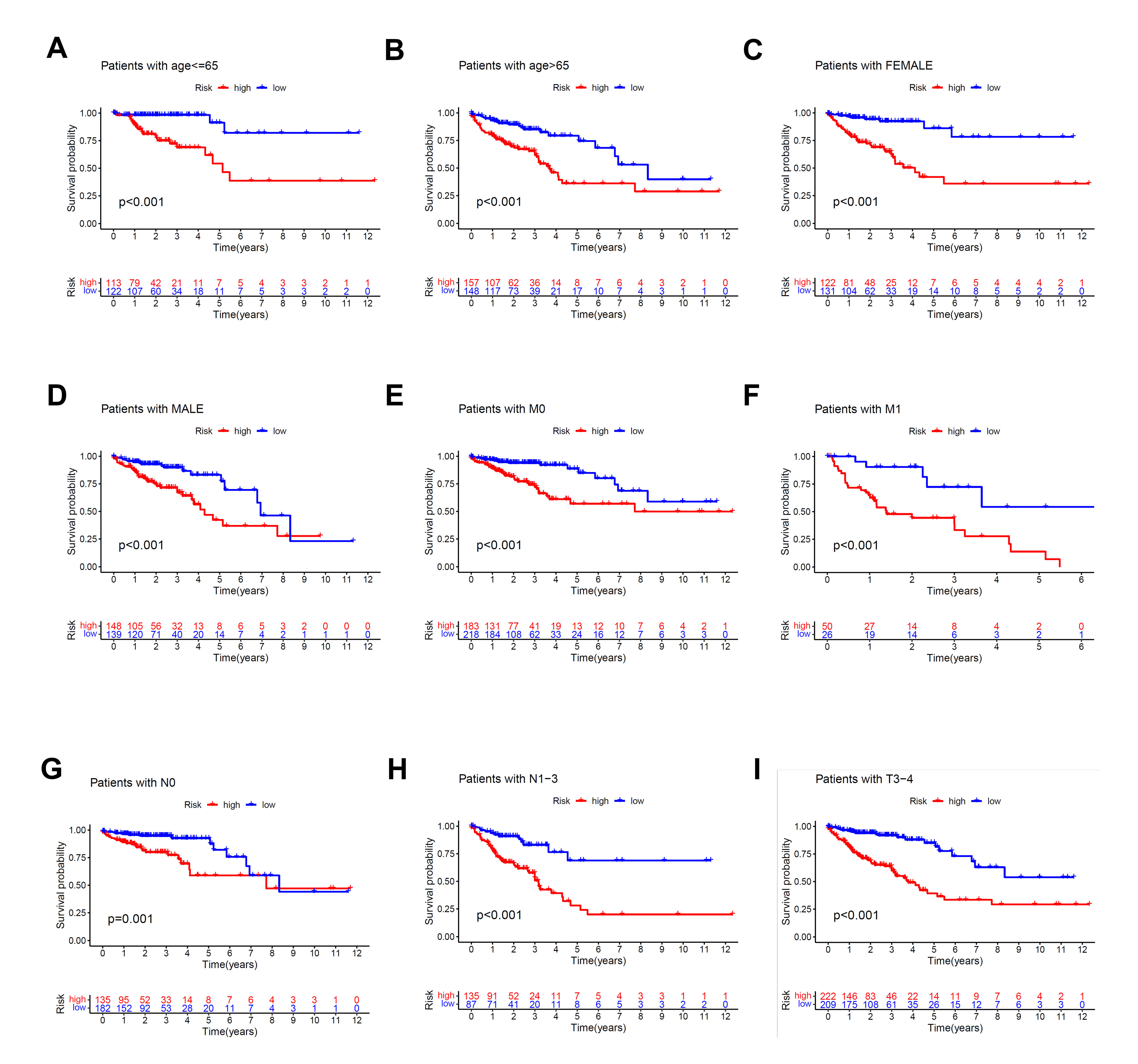

Supplement: Supplementary file 3 — Additional file 3: Figure S3. Prognostic value of the model based in subgroups including age, gender, stage, T stage, N stage, M stage on Kaplan Meier survival analysis. [file 12935_2023_3025_MOESM3_ESM.jpg]

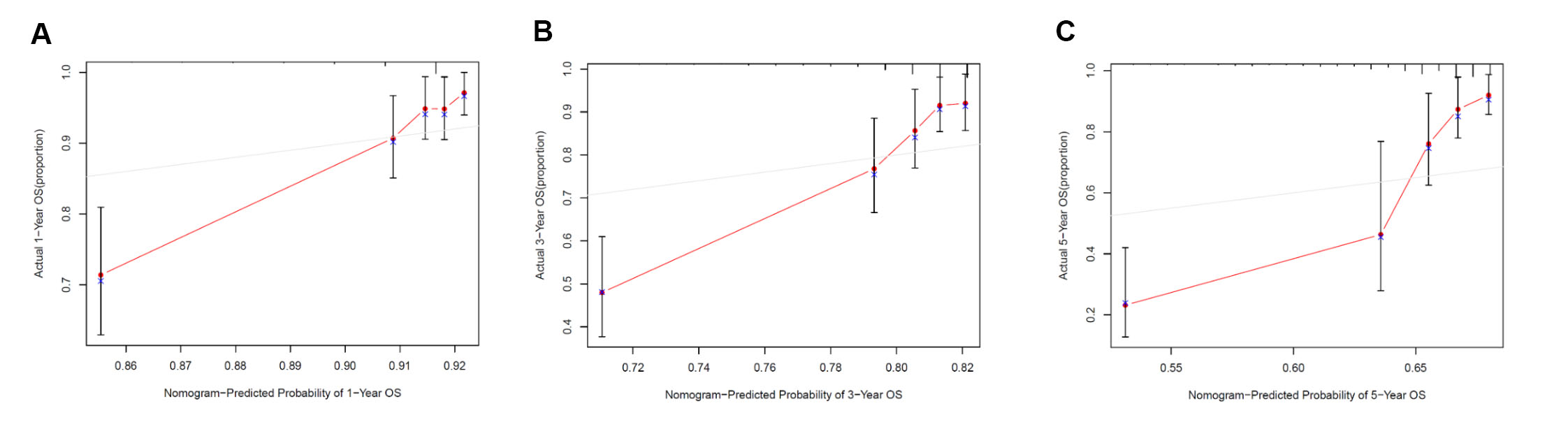

Supplement: Supplementary file 4 — Additional file 4: Figure S4. The calibration curve of the 1-, 3-, and -5 years overall survival between the actual observation and nomogram prediction. [file 12935_2023_3025_MOESM4_ESM.jpg]
